# Polycomb complex protein BMI-1 promotes invasion and metastasis of pancreatic cancer stem cells by activating PI3K/AKT signaling, an *ex vivo*, *in vitro*, and *in vivo* study

**DOI:** 10.18632/oncotarget.7078

**Published:** 2016-01-30

**Authors:** Min-Cong Wang, Min Jiao, Tao Wu, Li Jing, Jie Cui, Hui Guo, Tao Tian, Zhi-ping Ruan, Yong-Chang Wei, Li-Li Jiang, Hai-Feng Sun, Lan-Xuan Huang, Ke-Jun Nan, Chun-Li Li

**Affiliations:** ^1^ Department of Medical Oncology, The First Affiliated Hospital of Xi'an Jiaotong University, Xi'an, Shaanxi Province, People's Republic of China

**Keywords:** cancer stem cells, BMI-1, invasion, metastasis, pancreatic cancer

## Abstract

Cancer stem cell theory indicates cancer stem cells are the key to promote tumor invasion and metastasis. Studies showed that BMI-1 could promote self-renew, differentiation and tumor formation of CSCs and invasion/metastasis of human cancer. However, whether BMI-1 could regulate invasion and metastasis ability of CSCs is still unclear. In our study, we found that up-regulated expression of BMI-1 was associated with tumor invasion, metastasis and poor survival of pancreatic cancer patients. CD133+ cells were obtained by using magnetic cell sorting and identified of CSCs properties such as self-renew, multi-differentiation and tumor formation ability. Then, we found that BMI-1 expression was up-regulated in pancreatic cancer stem cells. Knockdown of BMI-1 expression attenuated invasion ability of pancreatic cancer stem cells in Transwell system and liver metastasis capacity in nude mice which were injected CSCs through the caudal vein. We are the first to reveal that BMI-1 could promote invasion and metastasis ability of pancreatic cancer stem cells. Finally, we identified that BMI-1 expression activating PI3K/AKT singing pathway by negative regulating PTEN was the main mechanism of promoting invasion and metastasis ability of pancreatic CSCs. In summary, our findings indicate that BMI-1 could be used as the therapeutic target to inhibiting CSCs-mediated pancreatic cancer metastasis.

## INTRODUCTION

Pancreatic cancer remains one of the most rapidly progressive and lethal malignancies in the world, with a mortality rate that almost equals its incidence. Over 80% of patients present with an unresectable primary tumor and distant metastasis at the time of diagnosis [1]. Moreover, once pancreatic cancer is diagnosed, the 1- and 5-year relative survival rates are 28% and 7%, respectively [2]. Because pancreatic cancer responds poorly to radiation and chemotherapy, surgical resection offers the only chance of cure at present. Surgical resection has been shown to increase patient survival by 10 months [3], but the majority of patients who undergo surgical resection still experience recurrence. To improve prognosis of patients with pancreatic cancer, it is essential to progress more effective treatments.

It is widely accepted that cancer is a disease of stem cells. Cancer stem cells (CSCs) have abilities of self-renew, multi-differentiation and tumor formation. Increasing experimental evidence supports that CSCs could stimulate growth, invasion, distant metastasis and relapse of many human cancers including pancreatic cancer [4]. Pancreatic CSCs have been isolated and studied since 2007. CD133 was identified as CSCs maker of pancreatic cancer, and associated with tumor invasion and metastasis [5-9].

Oncogenic BMI-1(B-lymphoma Moloney murine leukemia virus insertion region-1) belongs to the Polycomb group (PcG) family. Overexpression of BMI-1 could stimulate malignant transformation, proliferation, invasion, distant metastasis and was associated with poor patient survival in various human cancers, including pancreatic cancer [10-14]. For example, Song et al. reported that BMI-1 overexpression aggravated lymph node metastasis of pancreatic cancer [12]. BMI-1 was also up-regulated in pancreatic cancer cell lines and increased tumor cells invasion in vitro [11, 15, 16]. Recently, BMI-1 was identified to promote self-renewal, differentiation and tumor formation of CSCs and it was an important “switch” to maintain stem cells properties [17-20]. Moreover, BMI-1 was highly enriched in CD133+ glioblastoma stem cells [19].

To assess the potential role of BMI-1 in regulation of invasion and metastasis ability of pancreatic CSCs and the underlying mechanism, we firstly investigated the association of BMI-1 and CSCs maker CD133 with clinicopathological parameters and survival of pancreatic cancer patients. We then knocked down BMI-1expression in pancreatic CSCs to assess the effect on regulation of tumor invasion and metastasis in vitro and in vivo. After that, we explored the underlying molecular mechanism. Our results indicated that BMI-1 was a promising therapeutic target to inhibiting CSCs-mediated pancreatic cancer metastasis.

## RESULTS

### BMI-1 and CSCs marker CD133 expression promote tumor invasion, metastasis and poor survival of pancreatic cancer patients

**Figure 1:**
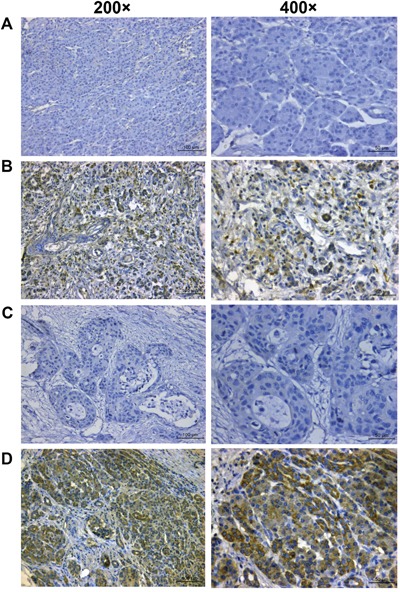
Immunohistochemical detection of BMI-1 and CD133 expression in pancreatic cancer tissues **A.** Negative staining of BMI-1. **B.** Positive staining of BMI-1. **C.** Negative staining of CD133. **D.** Positive staining of CD133. Images captured at x200 or x400 magnification.

Expression of BMI-1 and CSCs marker CD133 was assessed by using immunocytochemistry in 83 pancreatic cancer patients. Positive staining was indicated by brown granules. BMI-1 was localized in the nucleus and detected in 35 of the 83 tumor samples (42.2%). CD133 was localized mainly in the cell membrane and detected in 48 of these 83 tumors (57.8%) (Fig. 1). However, these two proteins were most negative in the corresponding distant non-tumor tissues.

We then assessed the association of BMI-1 or CD133 expression with clinicopathologic features of pancreatic cancer patients (Table 1). In brief, CD133 expression was associated with tumor AJCC stages and T stage, while BMI-1 expression was associated with tumor AJCC stages, T stage and lymphatic metastasis. Combining BMI-1and CD133 expression, we obtained the following two groups: CD133+/BMI-1+ and others (CD133+/BMI-1-, CD133-/BMI-1+, CD133-/BMI-1-). Notably, compared with the other combinations, the co-expression of CD133 and BMI-1 proteins was significantly associated with tumor AJCC stages, T stage and the lymphatic metastasis. These data suggested that CD133+ pancreatic CSCs and BMI-1 promoted tumor invasion (T stage) and lymphatic metastasis of pancreatic cancer.

**Table 1:** Association of gene expression with clinicopathological features of pancreatic cancer patients

Characteristic	No. of patients (%)	CD133		P-value	BMI-1		P-value	CD133 and BMI-1		P-value
		Negative	Positive		Negative	Positive		Others	Both positive	
Age (years)				0.953			0.953			0.161
≤60	43(51.8)	18	25		25	18		32	11	
>60	40(48.2)	17	23		23	17		24	16	
Gender				0.788			0.859			0.986
Male	46(55.4)	20	26		27	19		31	15	
Female	37(44.6)	15	22		21	16		25	12	
Site				0.206			0.206			0.891
Head and neck	47(56.6)	17	30		30	17		32	15	
Body and tail	36(43.4)	18	18		18	18		24	12	
Tumour size				0.255			0.822			0.084
≤3.5cm	32(38.6)	11	21		19	13		18	14	
>3.5cm	51(61.4)	24	27		29	22		38	13	
Tumour differentiation				0.409			0.409			0.831
Well/Moderate	54(64.9)	21	33		33	21		36	18	
Poor	29(35.1)	14	15		15	14		20	9	
Stage (AJCC)				0.010*			0.017*			0.004*
I-II	67(80.7)	33	34		43	24		50	17	
III-IV	16(19.3)	2	14		5	11		6	10	
T stage				0.030*			0.002*			0.001*
pT1-T2	43(51.8)	23	20		32	11		36	7	
pT3-T4	40(48.2)	12	28		16	24		20	20	
Lymph nodes				0.663			0.008*			0.029*
Negative	45(54.2)	18	27		32	13		35	10	
Positive	38(45.8)	17	21		16	22		21	17	
Metastases				0.813			0.189			0.094
Negative	72(86.7)	30	42		44	28		51	21	
Positive	11(13.3)	5	6		4	7		5	6	

Remarkably, there was significant association between expression of CD133 and BMI-1. CD133+ pancreatic cancer showed stronger BMI-1 expression than CD133- cases (Table 2). Co-expression (CD133+/BMI-1+) was detected in 27 of the 83 tumors (32.5%). Among the 48 patients with positive CD133 expression, 56.3% (27/48) showed positive BMI-1 expression. These data indicated that BMI-1 was up-regulated in CD133+ pancreatic CSCs.

**Table 2:** Association of CD133 with BMI-1 expression in pancreatic cancer tissue specimens

CD133	BMI-1		r	P-value
	Negative	Positive		
Negative	27	8		
Positive	21	27	0.334	0.002*

**Figure 2:**
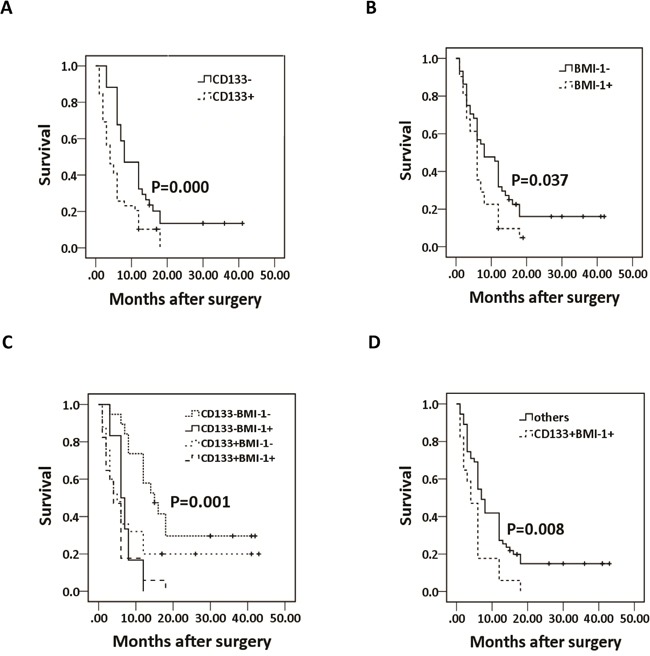
Kaplan-Meier survival curve analyses of survival of patients stratified by CD133 and BMI-1 expression using the log-rank test **A.** CD133 expression; **B.** BMI-1 expression; **C.** comparison of CD133-/BMI-1-, CD133+/BMI-1-, CD133-/BMI-1+ and CD133+/BMI-1+-; **D.** CD133+/BMI-1+ compared with other expression combinations. “+”represents the patients were alive until June 2014.

All of these 83 patients were followed up until June 2014. Of these 83 patients, 81.9% (68/83) died during the follow-up period and the survival time ranged from 1 months to >43 months, with a median survival time (MST) of 6.0 months. The MST of pancreatic cancer patients with CD133+ and CD133- was 4.0 and 8.0 months, respectively (Fig. 2A), and patients with BMI-1+ and BMI-1- was 6.0 and 8.0 months, respectively (Fig. 2B). We then analyzed the association of expression groups (CD133-/BMI-1-, CD133+/BMI-1-, CD133-/BMI-1+ and CD133+/BMI-1+) with survival of pancreatic cancer patients. The MST were 15.0 months (CD133-/BMI-1- patients), 5.0 months (CD133+/BMI-1-), 6.0 months (CD133-/BMI-1+) and 3.0 months (CD133+/BMI-1+) (Fig. 2C). Moreover, pancreatic cancer patients with co-expression CD133 and BMI-1 (CD133+/BMI-1+) tended to have poorer outcomes than those with other expression combinations (Fig. 2D).

We then grouped significant factors to analyze prognostic factors using log-rank test and revealed that age, tumor differentiation, AJCC stage, lymph node metastasis, distant metastasis, CD133, BMI-1, and co-expression of CD133 and BMI-1 were all significant prognostic indicators for overall survival of pancreatic cancer patients (P<0.05, Table 3). According to the results of the Cox's multivariate analysis of these factors, the predictive ability of tumor differentiation, AJCC stage, distant metastasis, CD133, BMI-1, and co-expression of CD133 and BMI-1 were confirmed (P<0.05, Table 3). These results supported CD133 and BMI-1 proteins could be used as prognostic markers for pancreatic cancer patients.

**Table 3:** Univariate and multivariate analysis of survival of 83 patients with pancreatic cancer

Risk factors	Univariate	Multivariate		
	P-value	RR	95%CI	P-value
Age, ≤60 vs. >60 years	0.006*			0.763
Gender, male vs. female	0.24			
Site, Head and neck vs. Body and tail	0.15			
Tumor size, ≤3.5 vs. >3.5cm	0.55			
Tumor differentiation, well/moderate vs. poor	0.000*	1.896	1.077-3.337	0.027*
Stage (AJCC), I-II vs. III-IV	0.001*	3.614	1.228-10.64	0.020*
T stage, pT1-T2 vs. pT3-T4	0.27			
Lymph nodes, negative vs. positive	0.012*			0.26
Metastasis, negative vs. positive	0.010*	1.397	0.775-2.520	0.012*
CD133, negative vs. positive	0.000*	2.208	1.161-4.203	0.016*
BMI-1, negative vs. positive	0.000*	2.661	1.256-5.638	0.011*
Co-expression of CD133 and BMI-1, others vs. CD133+/BMI-1+	0.000*	3.523	1.365-9.089	0.009*

### CD133+ pancreatic cancer cells have the characteristics of CSCs

**Figure 3:**
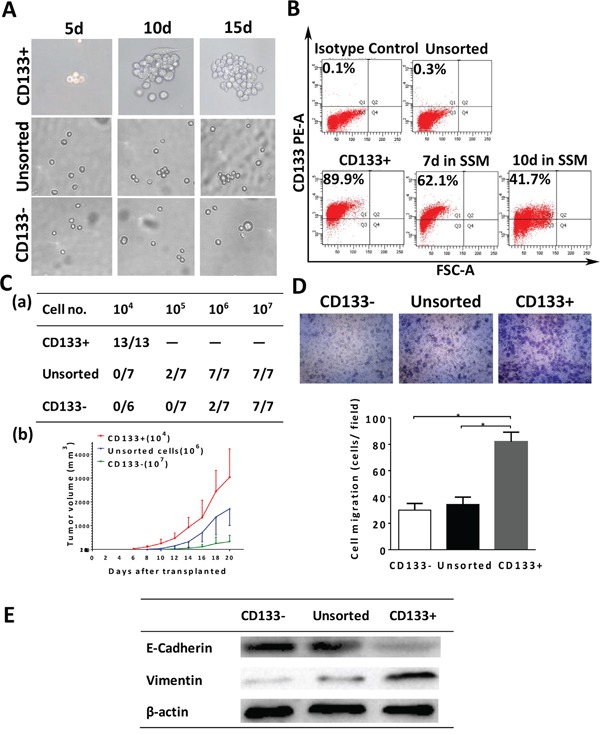
Characterization of CD133+ Colo357 cells **A.** Only CD133+ cells formed tumor spheres. **B.** Flow cytometric analysis of the purity and differentiation potential of CD133+ cells. **C.** (a) Tumor formation ability of CD133+, unsorted and CD133- cells; (b) Tumor growth rate of 10^4^ CD133+ cells, 10^6^ unsorted cells and 10^7^ CD133- cells in vivo. **D.** CD133+ cells increased invasion capacity compared to unsorted and CD133- cells (*P < 0.05 compared to CD133+ cells). **E.** Vimentin expression was increased and E-cadherin was decreased in CD133+ cells compared to unsorted and CD133- cells. SSM, serum-supplemented medium. Data were summarized as means ± SD.

We isolated CD133+ cells from Colo357 cells using CD133 magnetic cell sorting (MACS) and the purity of the sorted cells was validated by flow cytometry. As shown in Fig. 3B, the percentage of CD133+ cells in unsorted cells was 0.2±0.1% relative to the isotype control and enriched to 89.8 ±3.5% by MACS. Meanwhile, we also examined CD24, CD44 and ESA expression in Colo357 cells (Fig. S1A). The percentage of CD44+ or ESA+ cells was more than 90%, and CD24 was 33.5±2.2%.

CD133+ pancreatic cells were further characterized by a series of experiments since CSCs have the capacity of self-renew, multi-differentiation and tumor formation. First, we cultivated these CD133+, unsorted and CD133- cells in serum-free medium (SFM) to obtain tumor spheres formation. We found that CD133+ cells formed spheres and the size gradually increased, whereas unsorted and CD133- cells failed (Fig. 3A). To determine tumor differentiation potential, CD133+ cells were cultivated in serum-supplemented medium (SSM). After several days of culture, cells differentiated into large adherent cells. The percentage of CD133 expressing in CD133+ cells decreased to 62.1±3.0% after 7days and 41.7±2.1% after 10 days in SSM (Fig. 3B).

We then examined tumor formation abilities by implanted CD133+, unsorted and CD133- cells into the flank regions of nude mice. CD133+ cells exhibited significantly increased tumor formation abilities compared with unsorted and CD133- cells (Fig. 3C (a) and Fig. S2). CD133+ cells formed visible tumor in the shortest period of time for just 4 days, whereas CD133- cells took 10 days even more cells injected. All the mice which were injected 10^4^ CD133+ cells formed tumor. No tumor growth was evident unless at least 10^5^ unsorted cells or 10^6^ CD133- cells were injected. To examine the tumor growth rate when the tumor have initiated, we measured tumor volume of mice. As shown in Fig. 3C (b), the tumor growth rate of mice injected 10^4^ CD133+ cells was still higher than those injected 10^6^ unsorted or 10^7^ CD133- cells.

Studies showed that CSCs displayed enhanced metastatic potential in comparison to non-CSCs [21, 22]. Thus, we assessed invasion capacities of these CD133+ pancreatic cells using Transwell system and found that CD133+ cells had enhanced invasion ability than unsorted or CD133- cells (Fig. 3D). The epithelial-mesenchymal transition (EMT) could result in invasion and metastasis through down-regulation of E-cadherin and up-regulation of vimentin expression. We found that vimentin expression was increased, whereas E-cadherin expression was decreased in CD133+ cells (Fig. 3E). Taken together, CD133+ pancreatic cancer cells have the characteristics of CSCs.

### Expression of BMI-1 was up-regulated in pancreatic CSCs

**Figure 4:**
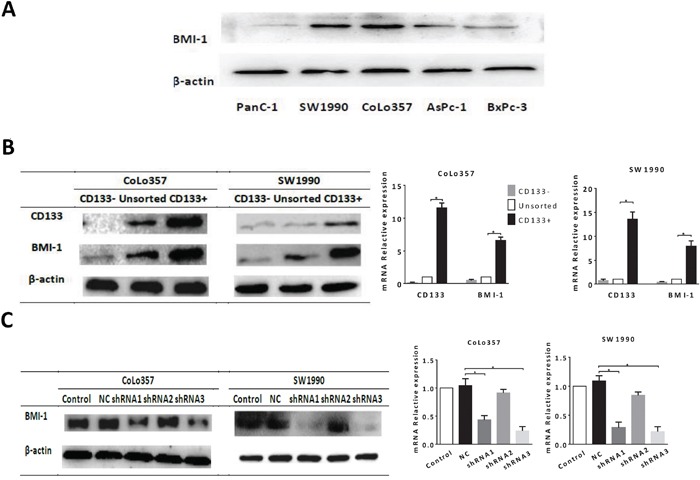
The expression of BMI-1 in pancreatic cancer cells and CSCs **A.** BMI-1 expression in five human pancreatic cancer cell lines. **B.** Level of BMI-1 protein and mRNA was up-regulated in CD133+ pancreatic CSCs isolated by using MACS (*P < 0.05 compared to unsorted). **C.** BMI-1 expression was knocked down by BMI-1-shRNA (*P < 0.05 compared to NC). MACS, magnetic cell sorting. Data were summarized as means ± SD.

We examined BMI-1 expression in five human pancreatic cancer cell lines, PanC-1, SW1990, Colo357, AsPc-1 and BxPc-3 using western blot (Fig. 4A). High levels of BMI-1 protein were noted in Colo357 and SW1990 cells. Thus, we used the two cell lines in the subsequent experiments. We examined BMI-1 expression in CD133+ Colo357 and SW1990 cells. Protein and mRNA expression of BMI-1 was higher in CD133+ cells than CD133− cells (Fig. 4B), while had no significant difference between CD24+ and CD24- cells (Fig. S1B). After that, we introduced shRNAs to knockdown BMI-1 expression in CoLo357 and SW1990 cells. Levels of BMI-1 protein and mRNA were decreased significantly by shRNA3 transfection (Fig. 4C), and thus, select shRNA3 for the following experiments.

### The BMI-1 promote pancreatic CSCs invasion and metastasis ability

**Figure 5:**
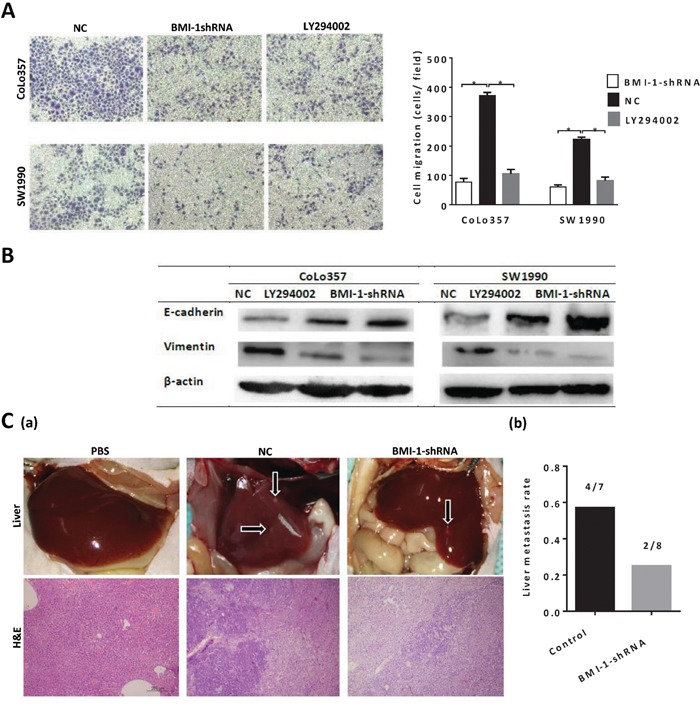
BMI-1 and activating PI3K/AKT singing pathway could promote invasion and metastasis capacity of pancreatic CSCs **A.** The invasion ability of CD133+ pancreatic CSCs significantly decreased by BMI-1-shRNA transfection or an inhibitor of PI3K/AKT singing pathway (*P < 0.05 compared to NC). **B.** E-cadherin expression was increased and vimentin was decreased by BMI-1-shRNA transfection or LY294002 treatment. **C.** (a) Representative images and H&E-stained sections of liver metastasis; (b) Quantitative data of liver metastasis detected by autopsy. Data were summarized as means ± SD.

We then assessed the effect of BMI-1 knockdown on regulating invasion ability of pancreatic CSCs. As shown in Fig. 5A, the invasion ability of CD133+ cells was significantly decreased by BMI-1-shRNA transfection compared to negative control shRNA (NC). Unexpectedly, we also found that invasion ability of CD133+ cells was also significantly decreased by LY294002, inhibitor of PI3K/AKT signaling pathway (Fig. 5A). E-cadherin expression was increased and vimentin was decreased by BMI-1-shRNA transfection or LY294002 (Fig. 5B), suggesting that the EMT was inhibited. The results revealed that BMI-1 or activating PI3K/AKT signaling pathway could promote pancreatic CSCs invasion.

To investigate whether BMI-1 is crucial for pancreatic CSCs metastasis in vivo, we performed an in vivo tumor cells metastasis assay. 1× 10^5^ CD133+ CoLo357 cells, transfected with negative control shRNA (NC) or BMI-1-shRNA, were injected into the caudal vein of nude mice (n=10). There were seven mice in NC group and eight in BMI-1-shRNA group alive after 8 weeks. Four out of seven mice developed liver metastasis in NC group, however, only two mice developed liver metastasis in BMI-1-shRNA group (Fig. 5C). The results suggested that BMI-1 was a key player to promote pancreatic CSCs metastasis.

### BMI-1 promote pancreatic CSCs invasion and metastasis by activating PI3K/AKT signaling

**Figure 6:**
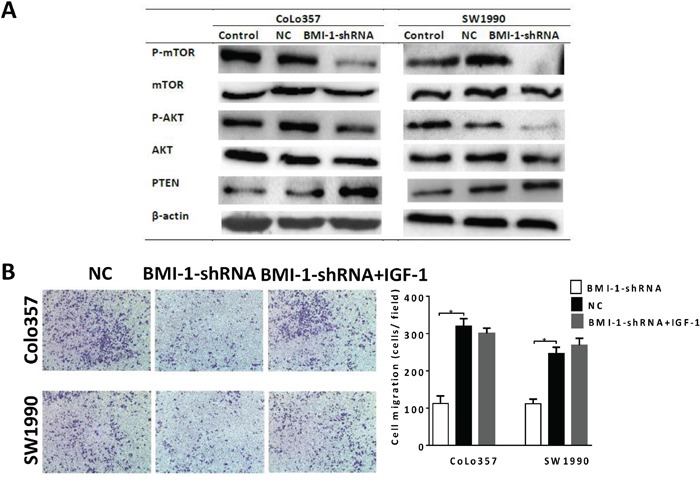
BMI-1 promoted invasion and metastasis ability of pancreatic CSCs by negative regulating PTEN and activating PI3K/AKT singing pathway **A.** Expression of P-AKT and P-mTOR was decreased, whereas PTEN expression was increased after knockdown of BMI-1. **B.** IGF-1 facilitated the invasive when abrogation of BMI-1 (*P < 0.05 compared to NC). Data were summarized as means ± SD.

To investigate whether BMI-1 regulated invasion and metastasis of pancreatic CSCs by activating the PI3K/AKT signaling pathway, we examined AKT and mTOR expression. As a transcriptional repressor, BMI-1 is unable to directly activate the PI3K/AKT pathway. It is well known that the tumor suppressor PTEN negatively regulates the PI3K/AKT signaling pathway, so we also examined PTEN expression. Expression of P-AKT and P-mTOR was decreased, whereas PTEN expression was increased after knockdown of BMI-1 using the BMI-1-shRNA (Fig. 6A).

IGF-1 is an activator of PI3K/AKT signaling pathway. When CoLo357 and SW1990 cells were treated with 50ng/ml IGF-1 for 0, 3, 6 and 12 h respectively, the expression of P-AKT and P-mTOR was significantly increased in 6h (Fig. S3). Then, we added 50ng/ml IGF-1 for 6 h to pancreatic CSCs which were transfected with BMI-1-shRNA and found that the invasion ability of pancreatic CSCs had no difference compared to NC ((Fig. 6B). The results further confirmed that BMI-1 promoted invasion and metastasis capacity of pancreatic CSCs by activating PI3K/AKT signaling pathway.

## DISCUSSION

Growing evidence suggests that CSCs could cause tumor relapse and metastasis [23, 24]. To date, the exact surface markers to distinguish pancreatic CSCs are still debatable. Li and colleagues firstly identified that CD44+/CD24+/ESA+ pancreatic cancer cells showed the stem cell properties, including self-renewal, multi-differentiation and tumorigenicity [25]. Other group found that CD133+ cells had higher tumorigenic and metastatic potential than CD44+/CD24+ cells, suggesting that CD133 might be a meaningful cell surface marker for pancreatic CSCs [9]. Furthermore, c-Met and ALDH1 (aldehyde dehydrogenase-1) were also identified as new makers for pancreatic CSCs [26-28]. In the current study, we used CD133 as the single biomarker to categorize pancreatic cancer cell population. CD133+ cells sorted from Colo357 cells were highly tumorigenic and displayed the capacity for self-renew and multi-differentiation. We also showed that CD133+ pancreatic cells had significantly stronger invasion capacity than CD133- cells, which were consistent with previous reports on CSCs from other types of cancer [8, 21, 29]. These results indicated that CD133 could be used as an independent and dependable CSCs marker in pancreatic cancer. It is well known that pancreatic cancer has the high rate of distant metastases and CSCs are the main reason. Our current study provided a novel molecular mechanism to explain how pancreatic CSCs gain their superior invasion and metastasis capability.

Numerous studies have suggested that increased CD133 or BMI-1 expression could lead to a significantly shorter overall survival in many human solid tumors. For example, previous meta-analysis showed that CD133 level was significantly correlated with lymph node metastasis and overall survival of NSCLC patients [30]. In the digestive system, CD133-positive gastric cancer and colorectal cancer patients had worse prognosis [31, 32]. BMI-1 is also related to poor prognosis of NSCLC, ovarian cancer, head and neck cancer and hematological malignancies [33-37]. In our study, we found that BMI-1expression was associated with AJCC stages, T stage and lymphatic metastasis, while CD133 was associated with AJCC stages and T stage. These properties of BMI-1 provide strong evidence for the role in linking pancreatic CSCs to invasion and metastasis. Furthermore, the median survival time of pancreatic cancer patients with BMI-1+ or CD133+ was significantly shorter than others. Multivariate analysis showed that tumor differentiation, AJCC stage, distant metastasis, CD133, BMI-1, and CD133 with BMI-1 all predicted poor survival of pancreatic cancer patients. In addition, overexpression of BMI-1 and CD133, taken together, will predict poor survival of pancreatic cancer patients.

EMT is known to be a central mechanism responsible for invasion and metastasis of human cancers. BMI-1 has been shown to be involved in EMT in cancer cells, such as endometrial cancer, oral cancer, lung cancer and nasopharyngeal cancer [4, 38-41]. Our current data showed that CD133+ pancreatic cells and CD133- cells had distinct patterns of BMI-1 expression. These data led us to focus on the role of BMI-1 in pancreatic CSCs. We found that tumor invasion and metastasis ability was significantly decreased in pancreatic CSCs in vitro and vivo by knockdown of BMI-1 expression, which was accompanied by increasing E-cadherin expression. These results suggest that BMI-1 is necessary for invasion and metastasis of pancreatic CSCs. However, the exact mechanism is unclear.

In the current study, we found that LY294002, an inhibitor of the PI3K/AKT signaling pathway, suppressed invasion of pancreatic CSCs. Then we examined protein expression of PTEN/PI3K/AKT signaling pathway and found that PTEN expression was increased, whereas P-AKT and P-mTOR levels were decreased after knockdown of BMI-1. These results suggest that BMI-1could activate PI3K/AKT signaling pathway by negative regulating PTEN in pancreatic CSCs. Insulin-like growth factor 1 (IGF-1) mediates various cellular processes, including proliferation, survival, and metabolism. The binding of IGF-1 to IGF-1 receptor leads to the activation of downstream signaling pathways, such as PI3K/AKT [42-45]. Our results showed knockdown of BMI-1 inhibited PI3K/AKT signaling and suppressed cells invasion. We observed that activation of PI3K/AKT signaling by IGF-1 facilitated the invasive when abrogation of BMI-1. Taken together, our findings indicate that BMI-1 promote invasion and metastasis ability of pancreatic CSCs by negatively regulating PTEN and activating PI3K/AKT signaling pathway.

The CSCs model has profound clinical implications and several dozen early-phase clinical trials aimed at CSCs are in progress [46]. Our study provides strong evidence that BMI-1 expression promote pancreatic CSCs invasion and metastasis, suggesting that BMI-1 could be a novel target for pancreatic CSCs. PTC-209 is a selective BMI-1 inhibitor. Kreso et al. showed that PTC-209 inhibited BMI-1 expression, and suppressed colorectal CSCs self-renew and tumorigenesis [47]. Thus, PTC-209 may be a promising treatment strategy for pancreatic CSCs, but future studies should be undertaken.

## MATERIALS AND METHODS

### Patients and tissue samples

A total of 83 pathologically confirmed surgical tissue specimens from pancreatic cancer patients were obtained from The First Affiliated Hospital, Xi'an Jiaotong University (Shanxi, China) between January 2009 and February 2013. Patient information was retrieved from their medical records and is shown in Table 1. Specifically, there were 46 males and 37 females with age between 34 and 78 years old (median age 60±10 years). None of the patients had undergone either chemotherapy or radiotherapy prior to the surgery. Tissue sections were reviewed by two experienced pathologists to verify histology assessment. Overall survival was defined as the time from surgery to mortality or was censored at the last known date alive. Prior informed consent was obtained and the study protocol was approved by the Ethics Committee of the Xi'an Jiaotong University.

### Immunohistochemistry

All formalin-fixed and paraffin-embedded tissue samples were sectioned at a thickness of 4 μm. The sections were dewaxed xylene and rehydrated in a graded ethanol series. The antigen retrieval was conducted using a vacuum -induced method in 0.01 M sodium citrate buffer for 2 min and allowed to cool down to the room temperature for about 20 min. Endogenous peroxidase activity was inhibited in 3% hydrogen peroxide/methanol for 15 min. After blocked in 1% normal goat serum, sections were incubated with a primary antibodies antibody against human CD133 (1:150, Millipore, Billerica, MA, USA) or BMI-1 (1:100 Abcam, Cambridge, MA, USA,) at 4°C overnight. Following incubation with a secondary antibody for 60 min and streptavidin-biotinylated horseradish peroxidase complex for 15 min at the room temperature, the sections were visualized by incubating with 3,3′-diaminobenzidine tetrahydrochloride (DAB) and counterstained in hematoxylin. After that, the sections were dehydrated in a graded series of ethanol, treated with xylene, and mounted in a synthetic resin.

CD133 and BMI-1 expression was scored as the sum of the intensity of staining and the proportion of positive cells. The staining intensity was evaluated as negative (0), weak (1), moderate (2), or strong (3). The percentage of positive cells was used to classify scores for specimens as follows: 0–5% (score 0); 5–25 % stained cells (score 1); 25–50 % stained cells (score 2); >50 % stained cells (score 3). Both positivity rate of cells and staining intensity were decided under a double-blind condition. The final expression score was ranged from 0 to 6, and a score of equal to or more than 4 was regarded as positive (high expression). The samples were evaluated under a microscope in five different fields at a magnification of 400×.

### Cell culture

Human pancreatic cancer cell lines SW1990, BxPc-3, AsPc-1 and Panc-1 were obtained from Hepatobiliary Surgery of The First Affiliated Hospital, Xi'an Jiaotong University (Shanxi, China). CoLo357 cell line was obtained from Hepatobiliary Surgery of Zhong Da Hospital of Dong Nan University (Jiangsu, China). SW1990 and Panc-1 cell lines were cultured in DMEM containing 10% fetal bovine serum (FBS) and 100 UI/mL penicillin–streptomycin. BxPc-3, AsPc-1 and CoLo357 cell lines were cultured in RPMI 1640 containing 10% FBS and 100 UI/mL penicillin–streptomycin. The sorted CD133+ pancreatic CSCs were cultured in DMEM/F12 supplemented with 20 ng/ml epidermal growth factor (EGF), 10 ng/ml basic fibroblast growth factor (bFGF) and B27 (1:50). All cell lines were grown at 37°C in a humidified atmosphere with 5% CO_2_.

### Western blot

Western blot was performed according to a previous study [48]. Briefly, cells were lysed with an RIPA cell lysis buffer (Wolsen Company, China) on ice. Equivalent amounts of protein samples was subject to SDS-PAGE gels (Millipore) and transferred on to Millipore membranes. The membranes were blocked for 1 h at the room temperature in 5% nonfat milk followed by the appropriate primary antibody overnight at 4°C [a rabbit mAb against human CD133 (Millipore, USA) and rabbit mAb against human BMI-1 (Abcam)]. The membranes were then washed with Tris-based saline-Tween 20(TBST) for 4 times for 10min each and then incubated with a secondary antibody at the room temperature for 1 h and exposed to x-ray films. To quantify the protein levels, films were scanned and analyzed using the lab works software.

### Real-time RT-PCR

Total RNA from cells was isolated using a Trizol reagent (Invitrogen, Carlsbad, CA, USA) and then reversely transcribed into cDNA synthesis following the manufacturers' protocol. qRT-PCR was performed using SYBR Green Master Mix (TaKaRa, Dalian, China). The PCR mixture (25 μl) contained 12 μl of 2xqPCR Master Mix, 1 μl cDNA, 1 μl of 10μM primers and 11μl of double distilled water. The sequences of primers used are shown in Table 4 and GAPDH was used as an endogenous control. qPCR was performed on an iQ5 Multicolor Real-Time PCR Detection System (Bio-Rad, Hercules, CA, USA). The reaction was in triplicate and consisted of a cycle of 95°C for 30 s followed by 40 cycles of amplification at 95°C for 5 s, at 60°C for 30 s and at 72°C for 30 s. The 2 [−DeltaDeltaC (T)] method was used to quantitate expression of each target gene [49].

**Table 4:** Oligonucleotide sequences of primers used in qRT-PCR

Gene		Sequence (5′—3′)
CD133	F	CATACCTAGGTCCCCGTCCG
	R	ATTTATGACCCGGCTTCTGGG
BMI-1	F	CTGGTTGCCCATTGACAGCG
	R	AAATCCCGGAAAGAGCAGCC
GAPDH	F	CCTCTGACTTCAACAGCGACAC
	R	TGGTCCAGGGGTCTTACTCC

### Cell sorting

CD133^+^ cell populations were isolated using a CD133 cell isolation kit (MiltenyiBiotec, Germany). First, cells were washed with phosphate buffered saline (PBS) and harvested by trypsinization and centrifuged, then resuspended in PBS. Then, cell solution was add 10 μL of PE-conjugated anti-human CD133 (130-090-853, MiltenyiBiotec), and incubated for 10 min in the dark in 4°C. After that, cells were washed with PBS, centrifuged and resuspended on PBS for addition of 20μL anti-PE MicroBeads (Miltenyi Biotec) per 1 × 10^7^ cells and incubated for 15 min in the dark in 4°C. The cells then magnetically separated with the auto-MACS Separator (MiltenyiBiotec) as described by the manufacturer's protocol. The resulted CD133+ cells were confirmed by using qRT-PCR, western blot and flow cytometric analysis.

### Tumor cell invasion assay

Tumor cell invasion assay was performed in the 24-well Transwell system with a polyvinyl polycarbonate filter of 8.0μm pore size (Millipore). The filter was coated with basement membrane Matrigel (BD Biosciences, Franklin Lakes, NJ, USA). 1 ×10^4^ cells in 200 μl serum-free medium were added to the upper chamber and 500μl of medium containing 10% FBS was placed in the lower chambers. The plates were then incubated for 24 h at 37°C in 5% CO_2_. The non-invaded cells were removed by a cotton swab and invaded cells were fixed and stained with crystal violet and then counted under a microscope for 10 random fields at a magnification of 100×. Each experiment was repeated at least three times.

### Nude mouse xenograft assay

Male severe combined immunodeficient (SCID) mice aged between 4 and 5 weeks were purchased from Xi'an Jiaotong University. Pancreatic CSCs (CD133+) were injected into mice using two different ways. In the first model, mice were subcutaneously implanted with CD133+, CD133- and unsorted cells. The cells were suspended in serum-free RPMI/Matrigel (BD Bioscience, San Jose, CA, USA) at (1:1 volume). Mice were monitored every two days for tumor growth and after 20 days, the mice underwent autopsy to assess tumor size and weight. Tumor volume (TV) was calculated using the formula TV = l (length) × w2 (width)/2. In the second model, to assess the effect of BMI-1 knock down on regulating metastasis of pancreatic CSCs xenografts, mice were divided into negative control and BMI-1-shRNA groups and 1× 10^5^ cells were injected into the caudal vein of mice. After 8w, the mice underwent autopsy and the harvested tissues were fixed in10% buffered formalin, embedded into paraffin and sectioned for further evaluation. This study was approved by the Institutional Ethics Committee.

### Statistical analysis

All analyses were performed using the SPSS 22.0 software (SPSS, Chicago, IL, USA) and data were expressed as mean ± standard deviation (SD) of at least three experiments. Asociations between immunostaining of CD133 and BMI-1 and clinicopathological variables of were evaluated using the χ2 test and Fisher's exact test. Kaplan-Meier curves were used to estimate association of CD133 and BMI-1expression with survival of patients and the equality of the two curves was compared by log-rank test. The log-rank test also was used for the univariate analysis, and a Cox proportional hazards regression model was used for the multivariate analysis of survival duration. Statistically significant differences between groups were determined by Student's t-test. A P<0.05 was considered statistically significant and all reported p-values were two-sided.

## SUPPLEMENTARY FIGURES


