# Supplementary material for: Polycomb complex protein BMI-1 promotes invasion and metastasis of pancreatic cancer stem cells by activating PI3K/AKT signaling, an ex vivo, in vitro, and in vivo study
Source: Oncotarget. 2016 Jan 30;7(8):9586–99. doi: 10.18632/oncotarget.7078 (PMC4891062; doi:10.18632/oncotarget.7078)
Supplement: Supplementary file 1 [file oncotarget-07-9586-s001.pdf]

SUPPLEMENTARY FIGURES

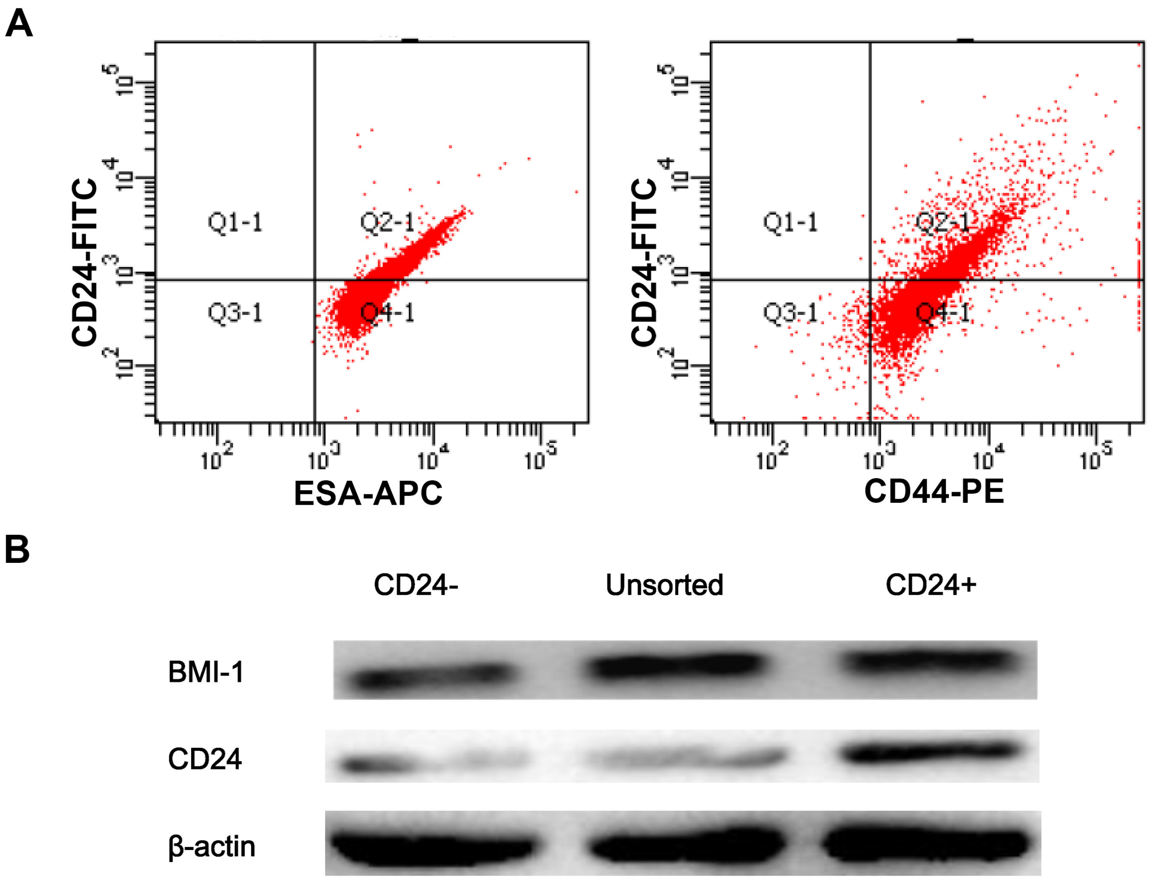

Supplementary Figure S1: BMI-1 expression in CD24+ Colo357 cells sorted by MACS. A. Flow cytometric analysis of CD24, CD44 and ESA expression in Colo357 cells. **B.** BMI-1 expression in CD24+ cells had no difference compared with CD24- or unsorted Colo357 cells.

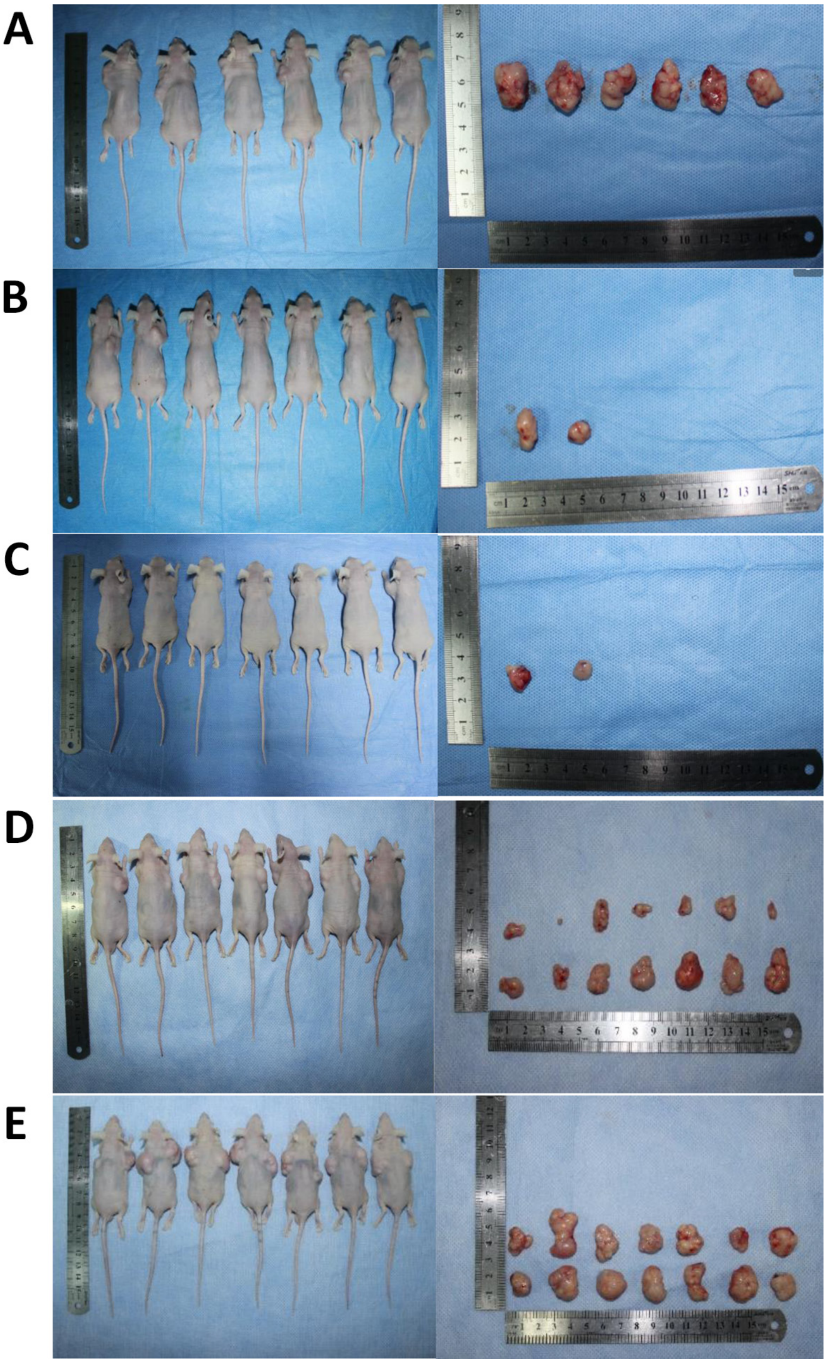

Supplementary Figure S2: The tumor formation ability of CD133+, unsorted and CD133- cells. **A.** 10^4 CD133+ cells and 10^4 CD133- cells were injected into SCID mice on the left and right flanks, respectively. **B.** Left, 10^5 CD133- cells; Right, 10^6 CD133- cells. **C.** Left, 10^4 unsorted cells; Right, 10^5 unsorted cells. **D.** Left, 10^7 CD133- cells; Right, 10^6 unsorted cells. **E.** Left, 10^7 unsorted cells; Right, 10^4 CD133+ cells. The upper tissue specimens were obtained from left flanks.

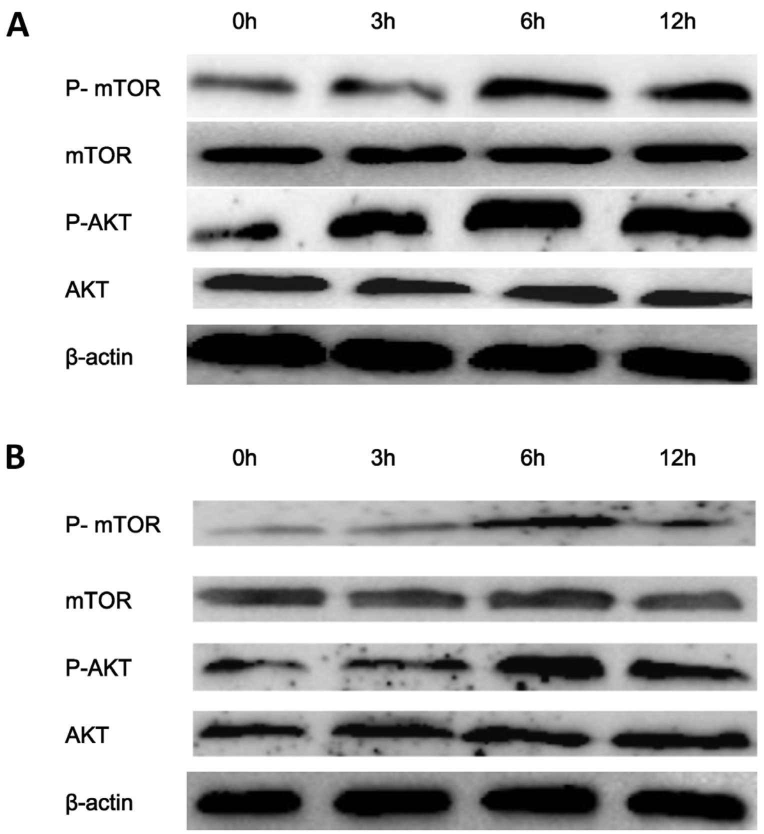

Supplementary Figure S3: Expression of PI3K/AKT singing pathway molecules at 0, 3, 6 and 12 hours after addition of IGF-1. A. Expression of PI3K/AKT singing pathway molecules of Colo357 cells. **B.** Expression of PI3K/AKT singing pathway molecules of SW1990 cells.
